# Clinical and socio-behavioral correlates of tooth loss: a study of older adults in Tanzania

**DOI:** 10.1186/1472-6831-6-5

**Published:** 2006-03-15

**Authors:** Irene A Kida, Anne N Åstrøm, Gunhild V Strand, Joyce R Masalu

**Affiliations:** 1 Centre for international health, UoB, Bergen, Norway; 2 Muhimbili University College of Health Sciences, Dar es Salaam, Tanzania; 3 Department of Odontology-Community Dentistry, UoB, Bergen, Norway; 4 Department of Odontology-Gerodontology, UoB, Bergen, Norway

## Abstract

**Background:**

Focusing 50 year olds and above, this study assessed the frequency, extent and correlates of tooth loss due to various reasons. Frequency and correlates of posterior occluding support was also investigated.

**Method:**

A cross-sectional household survey was conducted in Pwani region and in Dar es Salaam in 2004/2005. One thousand and thirty-one subjects, mean age 62.9 years participated in a clinical examination and completed interviews.

**Results:**

The prevalence of tooth loss due to any reason was 83.5 %, due to caries 63.4% and due to other reasons than caries, 32.5%. A total of 74.9% had reduced number of posterior occluding units. Compared to subjects having less than 5 teeth lost due to caries, those with 5 or more lost teeth were more likely to be females, having decayed teeth, confirming dental attendance and to be among the least poor residents. Compared to subjects who had lost less than 5 teeth due to reasons other than caries, those who had lost 5 or more teeth were more likely to be of higher age, having mobile teeth, being males, being very poor and to disconfirm dental attendance when having problems. Predictors of prevalence of tooth loss (1 or more lost tooth) due to various reasons and reduced number of occluding units followed similar patterns of relationships.

**Conclusion:**

The results are consistent with prevalence and extent of tooth loss due to caries and due to reasons other than caries being differently related to disease- and socio- behavioral risk indicators. Caries was the principle cause of tooth loss and molar teeth were the teeth most commonly lost.

## Background

The proportion of older people is growing faster than any other age groups throughout the world. By 2050, 2 billion people will be aged 60 years and above of whom 80% will be residents of developing countries [1]. Globally, poor oral health in older people is seen particularly as a high level of tooth loss, which in turn influences general health in terms of weight loss, eating problems and social handicaps related to appearance and communication [1].

Loss of permanent teeth can result from various events, either teeth are extracted by oral care providers or they are lost spontaneously due to progression of periodontal diseases or other events such as dental trauma [1]. Whilst dental caries and periodontal disease are the main reasons for tooth extractions, socio-economic-, behavioral- and attitudinal characteristics tend to influence the tooth retention profile of populations [2-7]. Epidemiological studies have shown that subjects of low income and education are more likely to be edentulous than their counterparts of higher income and education [8]. Tobacco use is a risk factor in tooth loss particularly in people having a high consumption over several years [1]. Recent surveys have shown higher frequency of tooth loss among adults in the industrialized countries than among their counterparts in developing countries, where access to dental care is limited [9-14]. Within many developing countries, urban dwellers and people of higher socio-economic status have easier access to dental care than their poor rural counterparts [15,16]. In Tanzania, since the government's health facilities are known to have shortage of essential equipments, many seek private facilities where charges for services are high and where no exemption of user fee system for the elderly is implemented [17]. Thus, one might expect affluent urban and poorer rural people to have the highest frequency of tooth loss and the highest rates of untreated oral diseases, respectively. Whereas industrialized countries spend 5–10% of their national public resources (GNP) on dental care each year, no budget is allocated to control for oral diseases in many developing countries [18]. This is noteworthy, considering that the burden of oral diseases is likely to grow in many developing countries because of transitions into unhealthy diets rich in sugar and increased consumption of tobacco products [19].

In Tanzania, information about the oral health status of the population is sketchy and mainly concerns children and adolescents. Reported epidemiological studies on tooth loss among older residents of mainland Tanzania, especially those living in rural areas, are very few [11,20]. A survey conducted as part of the NDHS (National Dental Health Survey) in the early 1980's, estimated frequencies of tooth loss of 83% (mean number of teeth missing 7.0) and 24% (mean number of teeth missing 0.8) due to caries and periodontal disease, respectively in adults 50 years and above [11]. In a more recent study of Tanzanian adults, Sarita [21] reported an average number of retained teeth ranging from 27 teeth in the youngest (20–29years) to 20 teeth in the oldest age group (above 60 years). Evaluating the function of the dentition, Sarita [12] reported a prevalence of shortened dental arches (SDA) (reduced number of posterior occluding units) of 15% in the adult population. In neighboring Kenya, Manji et al [9] reported that the majority of rural people retained most of their dentition up to the age of 65 years, whereas above 90% of > 55 year-olds had lost at least one tooth. Studies from other developing countries have reported a relatively high extent of tooth loss. A study of older individuals in Sri Lanka revealed a mean tooth loss of 20.7 SD10.7 among 60 year olds and above [10]. Susin et al [22] provided evidence of a mean tooth loss of 20 in Brazilian urban adults 60 years and older.

Since the independence in Tanzania in 1961, life expectancy at birth has been 50 years which places adults 35–40 yr and above in the elderly group of citizens [23]. Little is known with respect to the socio-demographic and behavioral correlates of the prevalence and extent of tooth loss among older adults and whether the rates of tooth loss in this age group have changed during the last two decades. Focusing community dwellers 50-years-old and above in urban and rural districts of Tanzania, this study aimed at assessing the frequency, extent and correlates of tooth loss due to dental caries and reasons other than dental caries. The frequency, correlates and functional consequences of having reduced premolar and molar occluding support were also investigated.

## Methods

### Study area

A cross sectional survey was conducted in Pwani region, Eastern Tanzania and in the capital city of Dar es Salaam from November 2004 to June 2005. According to the 2002 population and housing survey in Tanzania, Pwani region has the highest number of older people 65 years and above in the country (7%). Dar es Salaam and Pwani region have a total population size of 2.5 million and 889,154, respectively. The corresponding figures for population densities are 1,793 and 27 persons per square km. The districts have drinking water with fluoride content of about 1 mg F/L.

### Sampling and procedure

A stratified (disproportionate) two-stage cluster sample design with villages as the primary sampling unit was utilized. Villages were selected from two rural districts (Kibaha and Bagamoyo) and one urban (Kinondoni) district in Pwani and Dar es Salaam, respectively. To obtain a sample of older adults of mixed socio-economic background, 107 pure urban (N= 59688) villages and 96 pure rural villages (N = 26520) were listed in Kinondoni and in Kibaha/Bagamoyo, respectively. A sample size of 1200 adults in the defined age group was calculated assuming a prevalence rate of tooth loss (≥ 1 missing tooth) of 50%, a precision of 4% and a design effect of 2 [24]. At the first stage, 10 pure urban villages (n = 6290) and 10 pure rural villages (n = 3729) were selected by systematic random sampling from the district village population lists. At the second stage, a total of 60 households were selected by systematic random sampling from each village selected at the first stage. This involved randomly selecting the first household by spinning a bottle at the presumed center of each village to obtain a starting direction, listing on papers all household heads in the selected direction up to the boarder of the village, folding the paper and randomly picking one name. The next household would be one whose front door was nearest to the previous one. A household was defined as a group of people living, cooking and eating together. One person 50 years and above was enrolled per household. In case the household had several people in the targeted age group, one man and one woman were selected randomly. Over sampling of rural villages were implemented to achieve a sample size that was big enough to conduct stratified analyses. A village leader followed the data collectors through the village and traditional village protocol was observed ensuring a high response rate. A total of 511 (participation rate 85.2%) urban and 520 (participation rate 86.7%) rural subjects between 50 and 100 years (mean age: 62.9, SD = 10.6, men: 46.4%, no education: 44.7%), completed an extensive personal interview followed by a clinical examination. Only consenting subjects were included in the study. Exclusion criteria were presence of disease/conditions that might pose a health risk to the participant or that may interfere with the interview and clinical examination. Reasons for non-participation were refusals (n = 45), absence from household on the day of the interview n = 88). Subjects were excluded if they were ill or had a history of psychiatric problems (n = 23), were intoxicated with alcohol (n = 2), were too old (n = 7) or had beliefs in witchcraft (n = 4). Permission to carry out the study was approved by the Research and Publication Committee at Muhimbili University College of Health Sciences, regional and district administration authorities, village leaders and from the ethical research committee in Norway (REK VEST). In formed consent was obtained from all participating subjects.

#### Interview

A structured interview schedule was constructed in English and translated into Swahili before being administered in the field by two trained research assistants. Oral health professionals reviewed the interview schedule for semantic, experiential and conceptual equivalence. Sensitivity to culture and selection of appropriate words were considered. The interview schedule was piloted before administration. *Socio-demographics *were assessed in terms of place of residence, gender and age. *Level of education *was coded on a scale from (1) no education to (6) college/university. A dummy variable was constructed for analysis into (1) no education, (2) at least primary school education. *Family wealth *was assessed as an indicator of socio-economic status in accordance with a standard approach in equity analyses [25]. Household durable assets indicative of family wealth (e.g. bicycle, television, car, motor cycle) assessed as (1) available/in working condition, (2) not available/available but not in working condition, were included in a principle component analysis. The first component resulting from the analysis was used to divide households into four approximate quartiles of wealth status ranging from 1^st ^quartile (least poor) to 4^th ^quartile (most poor). *Frequency of dental attendance *during the previous 2 years – was coded (1) less than once and (2) once or more. *Reason for dental attendance *the previous 2 years was coded (1) when in problems (2) other reasons (including never go/go whether of not in problems). *Tobacco use *was assessed as (1) yes (2) no. A number of general health problems (e.g. high blood pressure) were assessed as (1) yes (2) no.

#### Clinical examination

One trained and calibrated dentist (IK) conducted all clinical examinations in a shaded area with natural daylight as the source of illumination and with an assistant recording the observations. Research assistants for recording were trained and calibrated before the main survey. Participants identified with problems that needed treatment were referred or advised to seek treatment from a nearest health care facility. Oral health education sessions were provided for all the participating subjects. *Plaque *was recorded initially using the mucosal – plaque index (MPS) [26] with the categories (1) no easily visible plaque (2) hardly visible plaque (3) moderate amount of plaque and (4) abundant amounts of confluent plaque. After cleaning of teeth by use of gauze, the dentition was inspected using disposable dental mirrors and probes, whereas cotton roles were used to control saliva. A full mouth clinical examination, including 3^rd ^molars was conducted. *Caries experience *was assessed in accordance with the criteria described by the World Health Organization, WHO [27]. A decayed tooth was recorded as present when a carious cavity was apparent on visual inspection supplemented by probing if required. Root tips were recorded as present and decayed tooth, if there was a caries lesion, while, they were scored other options, e.g. trauma, erosion, accordingly, when the tips had no caries lesion. If in doubt, no caries was recorded. A tooth was considered *missing due to caries *if there was a history of extraction because of pain and or the presence of cavity prior to extraction. *Teeth lost due to other reasons *were recorded separately and not included in the calculation of the DMFT score. *Prevalence of tooth loss due to any reason *was calculated with inclusion of edentulous people and defined as the percentage of individuals with ≥ 1 lost tooth. *Prevalence of tooth loss due any reason, due to caries and due to other reasons than caries *were recorded as (0) no teeth lost and (1) ≥ 1 tooth lost. *Extent of tooth loss due to caries and due to other reasons *were recorded as (1) ≥ 5 teeth lost (0) less than 5 teeth lost. *Tooth mobility *was assessed using a modified Miller's index [28] whereby the ends of two instruments were placed on either sides of the tooth and forces applied in bucco-lingual/palatal direction and scored as present or absent. An individual tooth mobility scores was defined as (1) 2 or more mobile teeth (0) less than 2 mobile teeth. *Functional premolar and molar occluding units *were counted based on existing natural tooth contacts between maxilla and mandible in the bilateral regions. The number of occluding pairs (with or without intact anterior region) was categorized into (1) complete posterior occluding support/10 functional occluding units, (2) reduced posterior occluding support/1–9 occluding units and (3) absence of bilateral occluding support. For analysis, a dummy variable was constructed yielding, (1) reduced occluding support (0–9 units) (0) and complete occluding support (10 units). The distribution of the POU variable supported this cut off point.

#### Reproducibility

Duplicate clinical examinations were carried out on a random sub-sample of the study subjects throughout the survey. Analysis performed on the duplicate examination recordings gave kappa statistics of 1.00 for missing teeth due to caries, decayed teeth and occluding support. Kappa statistics of 0.77, 0.79 and 0.51 were provided with respect to mobile teeth, tooth loss due to other reasons and plaque scores, respectively. These figures indicate a very good intra-examiner reliability (except for plaque) according to WHO [27].

#### Statistical analyses

Data were analyzed using SPSS version 13.0. Cross tabulation and chi-square statistics were used to assess bivariate relationships. Risk indicators for tooth loss frequency, extent of tooth loss and frequency of reduced premolar/molar support were estimated by stepwise logistic regression using the logit-model with 95% CI (confidence interval) given for the odds ratios indicating statistically significant relationship if both values were above or below 1. To adjust for the effect of the cluster design, re-analyses were conducted with STATA 9.0 using the svylogit command.

## Results

Table 1 gives the percentage distribution of participants' socio-demographic-, clinical-, and behavioral characteristics in urban Kinondoni and rural Kibaha/Bagamoyo districts. In addition to the data presented in Table 1, it was found that decayed teeth and mobile teeth were more prevalent in lower- than in higher family wealth groups (p < 0.001). Dental attendance patterns were more frequent in higher than lower family wealth groups (88.2% versus 68.7%, p < 0.001). Having 2 or more decayed teeth and 2 and more mobile teeth were most prevalent in females and males, respectively. Missing teeth due to caries and other reasons did not vary with the educational level of the participants (not in Table 1).

**Table 1:** Socio-demographic factors and oral health status indicators among older people in urban Kinondoni and rural Kibaha/Bagamoyo districts of Tanzania

	Kinondoni % (n)	Kibaha/Bagamoyo % (n)	p-value
Sex: *Male*	42.7 (218)	50.0 (260)	
*Female*	57.4 (292)	50.0 (260)	0.021
Age : *50–59 years*	50.3 (257)	37.9 (197)	
*60–69 years*	28.8 (147)	30.0 (156)	
*70+ years*	20.9 (105)	32.1 (167)	0.001
Wealth index: *1st quartile- least poor*	45.4 (232)	4.4 (23)	
*2*^*nd*^*quartile*	40.1 (205)	8.8 (46)	
*3rd quartile*	11.2 (57)	35.0 (182)	
*4th quartile- poorest*	3.3 (17)	51.7 (269)	0.001
Education: *none*	36.1 (184)	53.4 (277)	
*: at least primary school*	63.9 (325)	46.6 (242)	0.001
Tobacco use: *yes*	15.1 (77)	30.6 (159)	0.001
Reason dental attendance: *when problem*	87.3 (446)	71.4 (370)	0.001
Dental attendance: ≥ *one time*	21.1 (108)	24.2 (126)	0.231
High blood pressure: *yes*	26.2 (134)	6.7 (35)	0.506
Decayed teeth: ≥ *2 teeth*	46.0 (235)	55.4 (288)	0.050
Tooth mobility: ≥ *2 teeth*	16.2(83)	22.7 (118)	0.050
Brushing: *daily*	71.8 (367)	71.5 (372)	0.920
Plaque: *moderate/abundant*	44.1 (224)	47.2 (244)	0.175
Chewing: *only soft foods*	25.0 (129)	36.2 (189)	0.001

The prevalence of tooth loss (≥ 1 tooth lost due to any reason) in the study population, calculated with the inclusion of edentulous subjects (0.6% in urban and rural area) was 85.5% (mean tooth loss 6.1, SD= 6.4, mean tooth loss in affected subjects 7.1, SD = 6.3) in urban areas and 82.1% (mean tooth loss 5.9, SD= 6.6, mean tooth loss in affected subjects 7.2, SD = 6.5) in rural areas. Direct age standardization did not alter the crude urban rural difference in prevalence of tooth loss and there was no statistically significant difference by gender. The weighted prevalence and mean tooth loss in the total population of Dar es Salaam/Pwani region was 83.5 % and 5.8 teeth (SD = 6.4). Adults in the age groups 50–59 years, 60–69 years and 70+years had lost on average 5.5, 5.9 and 6.7 teeth due to any reason. The corresponding prevalence of tooth loss was 78.0%. 85.5% and 91.2%. A total of 63.4% (mean tooth loss 3.6) and 32.5% (mean tooth loss 2.4) had lost ≥ 1 tooth due to caries and due to other reasons, whereas 17.5%, 74.9% and 7.7% had respectively, 10-, 1–9- and 0 posterior occluding units.

**Figure 1:**
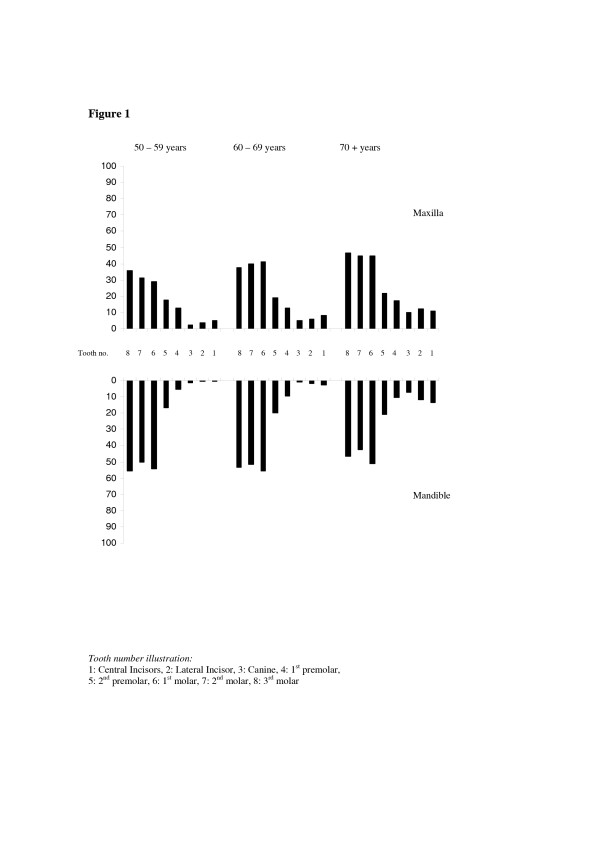
Percentage of tooth loss due to caries by tooth type and age group.

**Figure 2:**
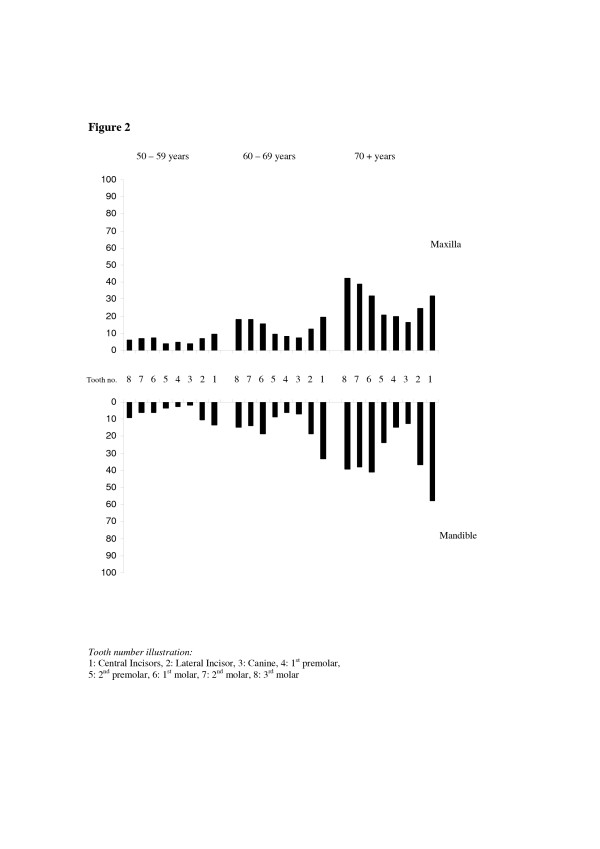
Percentage of tooth loss due to other reasons than caries by tooth type and age groups.

The distributions of tooth loss due to caries and due to other reasons according to tooth type and age groups are depicted in Figure 1 and Figure 2. Across all age groups, lower third and first molars were the teeth most frequently lost due to caries, whereas the lower central incisor was the tooth most frequently lost due to reasons other than caries. Table 2 shows the prevalence of subjects having lost ≥ 5 teeth and ≥ 1 tooth *due to caries *according to socio-demographic, behavioral and clinical factors and the corresponding odds ratios (OR) from multiple logistic regression analysis. Compared to subjects having less than 5 lost teeth, those having lost ≥ 5 were more likely to be females, of higher age, having higher family wealth, having decayed teeth and confirming dental attendance, and were less likely not to have high blood pressure. When controlling for all other variables in the model, a significant direct relationship occurred between age and extent of tooth loss due to caries (≥ 5 teeth). As shown in Table 2, the predictors of prevalence of tooth loss (≥ 1 lost tooth) followed a similar pattern of relationship as that shown for extent of tooth loss. The multiple logistic regression models explained 19.8 % (Nagelkerke's R^2 ^= .198, Model chi-square 155.390, df 10, p < 0.001) of the variance in the extent of tooth loss and 28.1% (Nagelkerke's R^2 ^.281, Model chi-square 236.631, df 10, p < 0.001) of the variance in prevalence of tooth loss due to caries. A statistical significant two-way interaction occurred with respect to decayed teeth by age upon extent of tooth loss. Separate regression models revealed that dental caries associated more strongly with tooth loss in younger than in older age groups. The odds ratios were 5.6 (95% CL 3.4–9.1), 2.2 (95% CL 1.2–3.9) and 1.6 (95% CL 0.9–2.8) in 50–59-, 60–69- and 70+year-olds, respectively

**Table 2:** Factors associated with having lost ≥ 5 teeth and ≥ 1 tooth due to caries. Chi square statistics, odds ratios (OR) and 95% Confidence limits (CL). Adjusted for use of tobacco (n = 1029).

	% (n) ≥ 5 teeth	OR (95% CL) (≥ 5 teeth)	% (n) ≥ 1 tooth	OR (95% CL) (≥ 1 tooth)
Age: *50–59 years*	28.6 (130)	1	65.2 (296)	1
*60–69 years*	32.0 (97)*	1.4 (1.1–2.0)	63.7 (193)	1.1 (0.8–1.6)
*70+years*	32.5 (89) *	1.7 (1.2–2.4)	60.2 (165)	1.1 (0.7–1.6)
Sex: *Male*	24.7 (118)	1	55.2 (264)	1
*Female*	35.8 (198)*	1.5 (1.1–2.0)	70.5 (390)*	1.7 (1.2–2.2)
Residence: *Urban*	37.4 (191)	1	71.4 (365)	1
*Rura*l	24.0 (125)	0.7 (0.5–1.1)	55.6 (289)*	0.5 (0.3–0.8)
Wealth index:				
*4*^*th*^*quart/poorest*	20.6 (53)	1	52.1 (134	1
*3*^*rd*^*quart*	27.1 (70)*	1.6 (1.0–2.9)	60.9 (157)	1.1 (0.5–1.8)
*2*^*nd*^*quart*	35.7 (97)*	1.8 (1.1–3.1)	70.2 (191)	1.3 (0.7–2.2)
*1*^*st*^*quart/least poor*	38.8 (94)	1.3 (0.8–2.1)	70.2 (170)*	1.1 (0.7–1.6)
Decayed: *0–1 teeth*	20.9 (106)	1	56.5 (287)	1
Decayed: *2–22 teeth*	40.2 (210)*	2.8 (2.1–3.8)	70.2 (367)*	2.1 (1.6–2.7)
Dental attendance: *Never*	11.7 (25)	1	27.7 (59)	1
Dental attendance: *When problems*	35.7 (291)*	3.2 (2.0–5.2)	72.9 (595)*	5.3 (3.6–7.7)
Dental attendance: *Never*	27.2 (217)	1	57.3 (457)	1
Dental attendance: ≥ *once*	42.3 (99)*	1.7 (1.2–2.3)	84.2 (197)*	2.8 (1.8–4.2)
High blood pressure: *yes*	46.2 (78)	1	78.1 (132)	1
High blood pressure: *No*	27.6 (238)*	0.6 (0.4–0.8)	60.6 (522)*	0.6 (0.3–0.9)

The total number in the different categories did not add up to 316 (≥ 5 teeth) and 654 (≥ 1 tooth) owing to missing values.

* p ≤ 0.05

Compared to those having lost less than 5 teeth due to other reasons than caries, subjects who had lost 5 or more teeth were more likely to be of higher age and to have mobile teeth, whereas they were less likely to be females, of higher family wealth and to attend a dentist when having problems (Table 3). A similar pattern of relationships occurred for the predictors of prevalence of tooth loss (≥ 1 lost tooth) due to other reasons. The complete models accounted for 27.3% (Nagelkerke's R^2 ^= .273, Model chi-square 174.964, df = 10, p < 0.001) of the variance in extent of tooth loss due to other reasons and 28.8% (Nagelkerke's R^2 ^= .288, Model chi-square 237.490, df = 10, p < 0.001) of the variance in prevalence of tooth loss due to other reasons.

**Table 3:** Factors associated with having lost ≥ 5 teeth ≥ 1 tooth due to reasons *other *than caries. Chi square and adjusted odds ratios (OR) and 95% confidence limits (CL).

	% (n) ≥ 5 teeth	OR (95% CL) ≥ 5 teeth	% (n) ≥ 1 tooth	OR 95% CL ≥ 1 tooth
*Age: 50–59 years*	6.8 (31)	1	20.0 (91)	1
*60–69 years*	15.5 (47)*	1.7 (1.1–2.8)	33.7 (102)*	1.4 (1.1–2.1)
*70+years*	28.8 (79)*	3.7 (2.3–6.0)	51.8 (142)*	3.1 (2.1–4.4)
Sex: *Male*	19.2 (92)	1	40.2 (192)	1
*Female*	11.8 (65) *	0.6 (0.4–0.9)	25.9 (143)*	0.5 (0.4–0.8)
Residence: *Urban*	9.8 (50)	1	24.3 (124)	1
*Rural*	20.6 (107)	1.2 (0.7–1.9)	40.6 (211)	1.3 (0.8–1.9)
Wealth index:				
*4*^*th*^*quart/poorest*	26.5 (68)	1	50.2 (129)	1
*3*^*rd*^*quart*	16.3 (42)	0.6 (0.3–1,4)	29.8 (77)	0.7 (0.4–1.3)
*2*^*nd*^*quart*	6.6 (18)*	0.3 (0.2–0.7)	22.4 (61)*	0.5 (0.3–0.9)
*1*^*st*^*quart/least poor*	12.0 (29)	0.8 (0.5–1.3)	28.1 (68)*	0.6 (0.3–0.9)
Dental attendance: *Never*	26.3 (56)	1	48.4 (103)	1
Dental attendance: *When problems*	12.3 (100)*	0.5 (0.3–0.8)	28.3 (231)*	0.5 (0.3–0.7)
Tooth mobility: *0–1 teeth*	9.5 (79)	1	24.5 (203)	1
Tooth mobility: ≥ *2 teeth*	38.8 (78)*	5.3 (3.5–7.9)	65.7 (132)*	5.4 (3.8–7.8)
Decayed: *0–1 teeth*	13.2 (67)	1	30.7 (156)	1
Decayed: *2–22 teeth*	17.2 (90)	1.2 (0.8–1.7)	34.2 (179)	1.0 (0.7–1.4)
Tobacco: *yes*	26.3 (62)	1	50.0 (118)	1
Tobacco: *no*	11.9 (95)	0.6 (0.4–1.0)	27.3 (217)*	0.6 (0.4–0.8)

The total number in the different categories did not add up to 157 (≥ 5 teeth) and 335 (≥ 1 tooth) owing to missing values.

* p ≤ 0.05

Table 4 depicts the adjusted ORs for reduced posterior occluding support. Number of decayed teeth, tooth mobility and age were the strongest predictors with odds ratios of 7.2, 3.0 and 2.7, respectively. Socio-demographics entered in the first step accounted for 8.1% (Nagelkerke's R^2 ^= .081, Model chi-square 51.4, df 7, p < 0.001). Entering behavioral and clinical variables raised the explained variance to 30% (Nagelkerke's R^2 ^= .301, Model chi-square 205.1, df = 12, p < 0.001). In a separate regression analysis, the ability to eat only soft/mashed foods varied systematically with reduced posterior occluding support whilst controlling for socio-demographic factors. The adjusted OR for having reduced chewing ability was 4.5 (95% CL 2.7–7.4) for subjects with 0–9 occluding pairs compared to their counterparts with 10 occluding pairs.

**Table 4:** Factors associated with reduced posterior occluding support. Multivariate analysis controlled for use of tobacco (n = 1023). Chi-square, odds ratios (OR) and 95% confidence limits (CL).

	% (n) 0–9 units	Adjusted OR (95% CL)
Age:*50–59 years*	78.0 (354)	1
*60–69 years*	82.5 (250)	1.3 (0.8–2.0)
*70+years*	90.0 (244) *	**2.7 (1.6–4.6)**
Sex: *Male*	79.3 (379)	1
*Female*	85.4 (472) *	**1.4 (1.0–2.1)**
Residence: *Urban*	87.9 (449)	1
*Rural*	77.3 (402)*	**0.3 (0.1–0.5)**
Wealth index:		
*4*^*th *^*quart/poorest*	79.4 (227)	1
*3*^*rd*^*quart*	78.7 (188)	1.0 (0.5–2.1)
*2*^*nd*^*quart*	86.9 (218)	1.1 (0.6–2.1)
*1*^*st*^*quart/least poor*	85.5 (218)	0.8 (0.5–1.3)
Decayed: *0–1 teeth*	71.1 (361)	1
Decayed: *2–22 teeth*	93.7 (490)*	**7.2 (4.6–11.1)**
Plaque: *no visible*	79.7 (444)	1
Plaque: *moderate/abundant*	85.7 (401) *	**1.5 (1.0–2.2)**
Tooth mobility: *0–1 teeth*	81.1 (665)	1
Tooth mobility: ≥ *2 teeth*	92.5 (186) *	**3.0 (1.6–5.5)**
Dental attendance: *Never*	71.4 (152)	1
Dental attendance: *in problems*	85.5 (694) *	**2.3 (1.2–3.5)**
Frequency attendance: *Never*	80.4 (644)	1
Frequency attendance: ≥ *once*	89.7 (210) *	**2.1 (1.2–3.5)**

The total number in the different categories did not add up to 851 owing to missing values *

p ≤ 0.05

## Discussion

The subjects investigated in this study experienced tooth loss that is similar to what has been observed decades ago in Tanzania and neighboring country, Kenya. [9,11]. It contrasts markedly with findings of much more extensive tooth loss in Sri Lanka, USA and Brazil [10,13,22]. Compared to the mean tooth loss of 5.9 teeth estimated for Tanzanians 61–69 year olds, recent surveys of the US and Brazilian populations have reported means of 13.2 and 18.1 teeth lost in comparable age groups [13,22]. Findings of the present study showed that 94.5%, 88.1% and 72.3% of the 50–59-, 60–69- and 70+year olds had retained 20 teeth or more. It appears that in this community-based sample of adults, the FDI recommended goal of 50% of individuals 65 years and older having ≥ 20 teeth are within reach [29]. Contrary to many previous studies, the estimates presented here are not adjusted for teeth indicated for extraction. Although information on caries severity was not available, a substantial unmet treatment need was reflected in the DT component constituting 70.5% of the total DMFT score. Thus, it is uncertain whether the FDI goals had been within reach if teeth indicated for extraction were accounted for. A previous survey of the Tanzanian population with comparable demographics to the present study population, revealed a figure for tooth loss due to caries that was similar to the present rate of overall tooth loss (83%) and higher than the present rate of tooth loss due to dental caries (63%) [11]. Sarita et al [21] reported a higher frequency of tooth loss among Tanzanian adults than what was obtained in this study. Based on the present results, tooth loss due to caries seems to have declined since mid 1980's among people 50 years and above in Tanzania. However, the difference in rates of tooth loss observed in the present and previous studies of Tanzanian older adults might be attributed to differences in study design and the characteristics of the study populations involved.

Both the prevalence and extent of tooth loss due to reasons other than caries increased sharply with increasing age in multiple logistic regression analysis. The presence of a positive relationship between age and tooth loss is in agreement with some other investigations, but at variance with others [6]. Consistent with results from previous studies, the present one revealed that caries was the major cause of tooth loss across the age groups investigated [9,11,30]. After adjusting for covariates, females and males were most likely to experience tooth loss due to caries and due to other reasons, respectively. Greater tooth loss in women than in men has been reported in many countries, although the reason for this gradient is still unclear [2,22]. In this study, women had experienced more decayed teeth but less tooth mobility than men and they attended dentists more frequently. Thus, the greater number of teeth lost due to caries in women appears to be related to dental caries experience and use of dental care services. Other studies have implicated periodontal disease as the leading cause of tooth loss as well as a higher prevalence of edentulous subjects in males compared to females [5].

It was documented for this sample that when compared to their less poor counterparts, the poorest subjects were more likely to experience dental caries, mobile teeth and teeth lost due to other reasons than caries. On the other hand, they were less likely to experience tooth loss due to caries and to seek dental care in response to oral problems. Findings from previous studies suggest that subjects of higher education and those who are wealthier in terms of economic status tend to have the lowest risk for tooth mortality [1,8,31,32]. It is probable that wealthy people afford preventive dental check-ups and conservative treatment that contribute to the retention of their teeth. In the present study, subjects who confirmed dental attendance frequently and when having problems had a higher frequency of tooth loss due to dental caries. This might be explained by a therapeutic rather than a preventive approach adopted by most dentists in Tanzania including the emergency oral health care, with extraction of teeth being the treatment offered for dental caries almost on a routine basis [16]. The reason why tooth mortality due to other oral problems was less common among dental attendees than among non-attendees is unclear. Previous studies in Tanzania have reported on few teeth with increased mobility even in individuals with extensive loss of supporting bone and on a relatively low frequency of teeth lost due to periodontal breakdown [20].

More poor subjects, although having the highest level of disease, seemed to be at lower risk for tooth loss due to caries and at higher risk of tooth loss due to other reasons because they did not attend the dental care system. With all variables in the model adjusted for and although the relationship was not linear, poorer subjects were still less likely to loose their teeth due to dental caries and more likely to loose their teeth due to other reasons compared to their wealthier counterparts. This might reflect social differences in the actual treatment offered, in the treatment opted to be received as well as behaviors and beliefs regarding the dental health care system in general. Although elderly people 60 years and above are exempted from user fees in Tanzania [33], most often dental clinics run out of necessary facilities and patients are requested to buy gloves, anesthetics etc in order to receive dental care. It should be noted that the sensitivity of the multivariate models was relatively moderate, suggesting that important characteristics of individuals loosing their teeth were not present in the analysis. Smoking status that was positively associated with tooth loss in this study most probably reflects other biological variables that were not included in the models [34].

It is evident that loss of occluding support not only associates with impaired chewing efficiency and inadequate nutrition [35,36] but also with other health problems such as lower extremity dynamic strength, agility and balance function in elderly adults [37]. Nevertheless, 10 occluding pairs from premolar to premolar have been recognized to satisfy function at a sub-optimal but acceptable level for older people [38]. The proportions of subjects with complete and reduced posterior occluding support in this study are not comparable to the figures pertaining to shortened dental arches reported by Sarita et al [12], due to different criteria. This study counted the number of posterior occluding pairs, an approach that has been used in some previous studies but not in many [2]. Consistent with earlier reports suggesting that difficulty with chewing food increases with decreasing number of occluding pairs, this study revealed that subjects with ≤ 9 occluding premolars/molars were about 4 times more likely to have chewing problems than their counterparts having complete posterior occluding support [2,12]. Locker [7] has argued for a need of information with respect to *when *tooth loss becomes problematic as well as for *whom*. The present findings indicate that having reduced posterior occluding support occurred most frequently in older subjects, females, urban residents, those experiencing un-restored caries, mobile teeth and assessable plaque and also in subjects who visited the dentist most frequently (Table 4).

The self-report method employed in the assessment of the causes of tooth loss are associated with uncertainty since their validity could not be verified by reports from dental records or health care workers having performed the extractions. Examining the distribution of dental caries within the dentition revealed however, a closer resemblance with the distribution of tooth loss due to caries than with the distribution of tooth loss due to other reasons across all age groups investigated [11]. Moreover, the finding that the mean number of teeth with untreated dental caries far exceeded the mean number of mobile teeth tends to confirm the general picture obtained from the interviews. In a detailed analysis of the pattern of periodontal breakdown of Tanzanian adults, Baelum [39] reported mandibular incisors to be among the teeth most affected with loss of attachment. As shown in Fig 2 and consistently with what has been reported previously in Tanzania and elsewhere, anterior teeth predominated among teeth lost due to other reasons, whereas posterior teeth predominated teeth lost due to caries [9,11,22]. A second limitation of this study was its cross-sectional design that might have weakened the association between dental disease and tooth loss. From this point of view, the interaction effect, with dental caries being a stronger predictor of tooth loss in younger rather than in older age group was not surprising.

## Conclusion

The results of this study are consistent with tooth loss prevalence, extent of tooth loss and reduced occluding support being a consequence of disease-, behavior-, and social related risk indicators and their interactions. Caries was the principle cause of tooth loss and molar teeth were most commonly lost. This is in accordance with other studies recently conducted in sub-Saharan Africa [40,41]. Tooth loss due to caries and tooth loss due to other reasons was closely but differently related to disease- and socio-behavioral factors. Not going to a dentist was associated with retention of carious teeth and with tooth loss due to reasons other than caries, whereas loss of occluding support impacted on chewing ability. Efforts to preserve more natural teeth of the ageing population should focus on the prevention and treatment of caries and periodontal diseases. Outreach emergency oral health care in Tanzania should be strengthened through education of dental care providers to equip them with means to treat and retain teeth.

## Competing interests

The author(s) declare that they have no competing interests.

## Authors' contributions

**IK**: Principal investigator, conceived of the study, designed the study, collected data, statistical analysis and manuscript writing

**AA**: Main supervisor, designed study, statistical analysis, manuscript writing

**GS**: Participated in design of study and manuscript writing

**JM**: Participated in design of study, data collection and manuscript writing

## Pre-publication history

The pre-publication history for this paper can be accessed here:


